# A 48-year-old man with fever, nauseous, vomiting, and dizzy: A CARE case report

**DOI:** 10.1097/MD.0000000000039015

**Published:** 2024-08-02

**Authors:** Xingbo Kou, Dinghao An

**Affiliations:** a Department of Thoracic Surgery, Shangluo Central Hospital, Shangluo, China; b Department of Neurology, Nanjing Drum Tower Hospital, Chinese Academy of Medical Sciences & Peking Union Medical College, Nanjing, China.

**Keywords:** : *Listeria*, meningoencephalitis, sepsis, muscle lesions, case report

## Abstract

**Rationale:**

*Listeria monocytogenes* (LM) is an important foodborne bacterium, and LM meningoencephalitis is rare in clinical practice, with poor prognosis in severe patients. It is prone to misdiagnosis in clinical practice. We first reported a case of severe LM meningoencephalitis with muscle lesions and evaluated the comprehensive condition.

**Patient concerns:**

A 48-year-old man had a fever and was admitted to the neurology department due to dizziness, nausea, and vomiting for 20 days.

**Diagnoses:**

LM meningoencephalitis complicated with muscle lesions.

**Interventions:**

We used moxifloxacin 0.4 g, qd, meropenem 2 g, q8h, and dexamethasone 10 mg, qd to reduce exudation and adhesion. Then due to consideration of side effects, we increased the dose of ampicillin by 2 g, q4h, stopped using meropenem and moxifloxacin, and turned to maintenance treatment with dexamethasone and ampicillin. We comprehensively managed his vital signs and physical organ functions, we also controlled some comorbidities. During the hospitalization period thereafter, we used intravenous anti-infection treatment with moxifloxacin 0.4 g, qd, ampicillin 0.5 g, q4h.

**Outcomes:**

Half a year later, the reexamination showed only protein elevation in cerebrospinal fluid and hydrocephalus in MRI. Afterward, the symptoms did not recur again. The patient recovered well after discharge.

**Lessons:**

LM meningoencephalitis complicated with lower limb muscle lesions is clinically rare. This report focuses on relevant treatment plans, which provide value for the examination and comprehensive management of patients with LM infection in the future.

## 1. Introduction

*Listeria monocytogenes* (LM) is an interanaerobic Gram-positive bacterium that is mainly transmitted through dairy, meat, food or semiprocessed foods. After entering the gastrointestinal tract, LM invade the intestinal mucosa and cause infection through the intestinal and blood–brain barriers. They are most likely to accumulate in the placenta and nervous system of mammals,^[1]^ leading to meningitis and even brain abscess. Although listeria infection is rare in humans (0.1–10 cases per 1 million), it has a fatality rate of up to 30% in cases with neurological lesions, making it the most serious foodborne bacterial infection.^[2]^ A retrospective case–control study showed that delayed treatment with appropriate antibiotics for more than 6 hours was associated with a 2.78-fold increase in mortality.^[3]^ Therefore, timely identification of the pathogen and initiation of appropriate antibiotic treatment is the key to a good prognosis. The population is generally susceptible to LM, especially neonates, pregnant women, and patients with immune deficiency. The main clinical manifestations include high fever, diarrhea, mental disorders, conscious disturbance, severe headache, vomiting, nausea, and other cranial hypertension symptoms. Physical examination indicates positive meningeal irritation sign. Its diagnosis mainly relies on blood, cerebrospinal fluid (CSF) culture, and next-generation sequencing (NGS) technology. It can be distinguished from viral, tuberculous and cryptococcal meningitis, but its clinical manifestations lack specificity compared with common purulent meningitis. And when patients have acquired immune deficiency syndrome (AIDS) or immune system diseases and tumors, differential diagnosis becomes particularly difficult. Studies on LM central nervous system infection have been reported in Europe, the United States, Australia, and Asia, accounting for approximately 4% to 16.5% of cases.^[4–9]^ In Central and Eastern European countries, there are few studies, limited to case reports. LM infection invading the central nervous system often causes isolated meningitis, encephalitis, or brainstem encephalitis. The classic affected areas are the dorsal brainstem, cerebellum, and fourth ventricle.^[10,11]^ In addition to the nervous system, the complications of the disease also involve other systems. However, there have been no previous reports of complications in the muscular system of LM meningoencephalitis. This is the first report of LM meningoencephalitis with space-occupying muscle lesions. And a detailed discussion was conducted on the experience and lessons learned from the diagnosis and treatment of this patient, aiming to provide certain value for the diagnosis and comprehensive management of patients with LM infection in the future.

## 2. Case presentation

A 48-year-old man was admitted to the neurology department of Nanjing Drum Tower Hospital due to dizziness, nausea, and vomiting.

Twenty days ago, the patient suddenly experienced symptoms of nausea, vomiting, and dizziness after lunch. They reported that on the day of onset, they tried intravenous acyclovir and dexamethasone in another hospital, but the symptoms did not improve. Fifteen days later, he began to have a fever, reaching a maximum of 39 °C. Two days after the fever, he went to another hospital for a blood routine examination. White blood cells were 19.52 × 10^9/L, neutrophils were 18.01 × 10^9/L, and blood culture showed *Listeria* infection. We then performed lumbar puncture, and the cells were 210 × 10^9/L, protein 1371.5 mg/dL, glucose 1.1 mmol/L, cranial magnetic resonance imaging (MRI) examination showed lesions in the right pontine and brachium pontis. Therefore, symptomatic treatment such as cephalosporin anti-infection and gastric protection was given. The symptoms improved and the patient returned home with intermittent fever for a few days. However, after 5 days, the patient developed a drowsy state, mental lethargy, unclear answers, and the condition gradually worsened. The patient was then sent to our hospital’s neurological intensive care unit by his family members. During the physical examination in our hospital, there was also sluggish light reflex and a stiff neck. On the day of admission, lumbar puncture was performed, along with CSF pathology and NGS examination, which indicated *Listeria* infection. CSF pathology (severe central nervous system inflammation, predominantly lymphocytes; no Cryptococcus or atypical cells detected. Leukocyte: 394/µL, Neutrophils: 16%, Lymphocytes: 72%, Monocytes: 11%, Plasma cells: 1%). NGS: (G + *Listeria*: Sequence Number: 3412, Relative Abundance: 95.63%; LM: Sequence Number: 2619; Q30:92.14). The details can be found in the Supplemental Digital Content, http://links.lww.com/MD/N340. In addition to continuous oxygen inhalation, electrocardiogram monitoring, intubation, enteral nutrition, etc, intravenous infusion of anti-infection treatment was initiated. We used moxifloxacin 0.4 g, qd, meropenem 2 g, q8h, and dexamethasone 10 mg, qd to reduce exudation and adhesion. On the second day of admission, blood routine examination was conducted, and only an increase in neutrophil ratio and normal procalcitonin levels were observed. The treatment plan was not changed. On the third day of admission, the patient’s consciousness still did not return to normal. A plain computed tomography (CT) scan of the head was normal. However, the patient complained of significant swelling and pain in the left leg. Due to long-term bed rest, we suspected it to be a venous thrombosis in the lower limb. However, to our surprise, deep vein ultrasound and D-dimer were normal, so we examined the lower leg. The temperature of the left leg decreased, the skin color and pulsation were normal, and there was tenderness. And after performing muscle ultrasound, we found occupying lesions in the left gastrocnemius muscle, and the lesion was widely distributed. There was a low echo area with a strip shape, covering an area of approximately 12.6 × 0.5 cm. And it was accompanied by thickening and swelling of the left plantar tendon, with a diameter of approximately 0.49 cm and reduced echo. Therefore, we increased the dose of ampicillin by 2 g, q4h, intravenous infusion. One day later, due to consideration of side effects such as secondary infection and worsening of muscle related lesions, we stopped using meropenem and moxifloxacin and turned to maintenance treatment with dexamethasone and ampicillin. When the patient was admitted for 10 days, his consciousness turned into drowsiness. Therefore, we conducted a follow-up examination of the CSF, and there was a slight improvement in cell counts, but the pressure was still low. Therefore, we did not stop the hormone and continued to use dexamethasone 10 mg qd. At the same time, we conducted a cranial MRI again, and the lesion around the pons was still large. Diffusion weighted imaging (DWI) showed a new abnormal high signal in the posterior horn of left lateral ventricle, and meningeal enhancement sign appeared. After discussion and consultation with the Department of Infectious Disease and Neurosurgery, the treatment strategy will not be changed temporarily. At the same time as using proton pump inhibitors to protect the stomach upon admission, the patient still experienced upper gastrointestinal bleeding twice during the course. After careful inquiry, we found that the patient had a history of duodenal ulcers before the onset of the disease, and gastroscopy electrocoagulation was performed to stop bleeding after consulting the Department of Gastroenterology. After 30 days of hospitalization, the patient returned to normal consciousness. At the end of the anti-infection treatment for 35 days, we reexamined the CSF and found that the pressure had returned to normal, at 125 cm H_2_O, with only an increase in protein. At the same time, we reexamined a cranial MRI and found that the core lesion in the brainstem had shrunk compared to before, yet with brain edema in the lateral ventricle. We requested further consultation from the infectious disease department. They recommended the addition of moxifloxacin. Taking into account the patient’s general condition, we decided temporarily not to reduce intracranial pressure through dehydrating. And upon admission for 45 days, a reexamination of the head CT scan revealed changes in hydrocephalus, which were also concentrated in the lateral ventricle. The patient had been hospitalized for 50 days, and his consciousness had returned to normal with normal blood routine. The patient requested to be discharged and allowed to continue intravenous infusion of ampicillin 2 g q4h for 1 month at the local hospital.

While carefully inquiring the medical history of the patient, considering the condition of the patient, we asked his family members, they clearly stated that they had no history of contact with soil or chemical toxins. Moreover, they had lived in the local area for a long time and had no history of tourism or contact with pasturing area personnel in the past 2 years. They did not keep pets or poultry at home. And they also said the patient had no insect bites areas in his skin and any injuries before the onset. He did not have drug use, or other bad habits that allow pathogens to enter the bloodstream. And the patient had no past history or family history of immune diseases, infectious diseases, or tumors. The family members reported that the patient has no special history of unclean eating. After the patient returned to consciousness, we asked the patient again about the aforementioned medical history, and the answers we obtained were consistent. During the hospitalization, we comprehensively managed his vital signs and physical organ functions, we also controlled some comorbidities such as hypertension, type 2 diabetes, metabolic syndrome, etc well.

After returning home, the patient developed fever due to COVID-19 infection, and was treated with physical cooling, ibuprofen and Lan Qin KouFuYe, a kind of traditional Chinese medicine. Half a month later, dizziness and mental lethargy appeared, and family members reported a slowdown in speech speed and slow response. Upon readmission, a neurological examination revealed increased muscle tone in the limbs and neck stiffness. On the day of admission, lumbar puncture examination showed high protein levels and slightly elevated cells, after excluding the COVID-19 related neurological complications, we thought it may be a recurrence of LM meningoencephalitis. Ultrasound of the gastrocnemius muscle lesions were similar to before, and muscle MRI scanning showed a significant increase in the external end of the gastrocnemius muscle with high signal pressure and no enhancement. The core lesion of the brachium pontis became smaller than before, and the high signal of the left posterior horn of lateral ventricular disappeared in DWI, in addition, hydrocephalus had improved a lot. The dynamic electroencephalogram was normal. We immediately initiated intravenous anti-infection treatment with moxifloxacin 0.4 g, qd, ampicillin 0.5 g, q4h. On the evening of the 17th day of admission, the patient developed chills and fever. We provided physical cooling and continued the original anti-infection treatment plan. Twenty four days later, a CSF reexamination was performed with all indicators improved. The infection and inflammation indicators were normal. The patient felt that his symptoms had improved and requested discharge.

**Figure 1.**
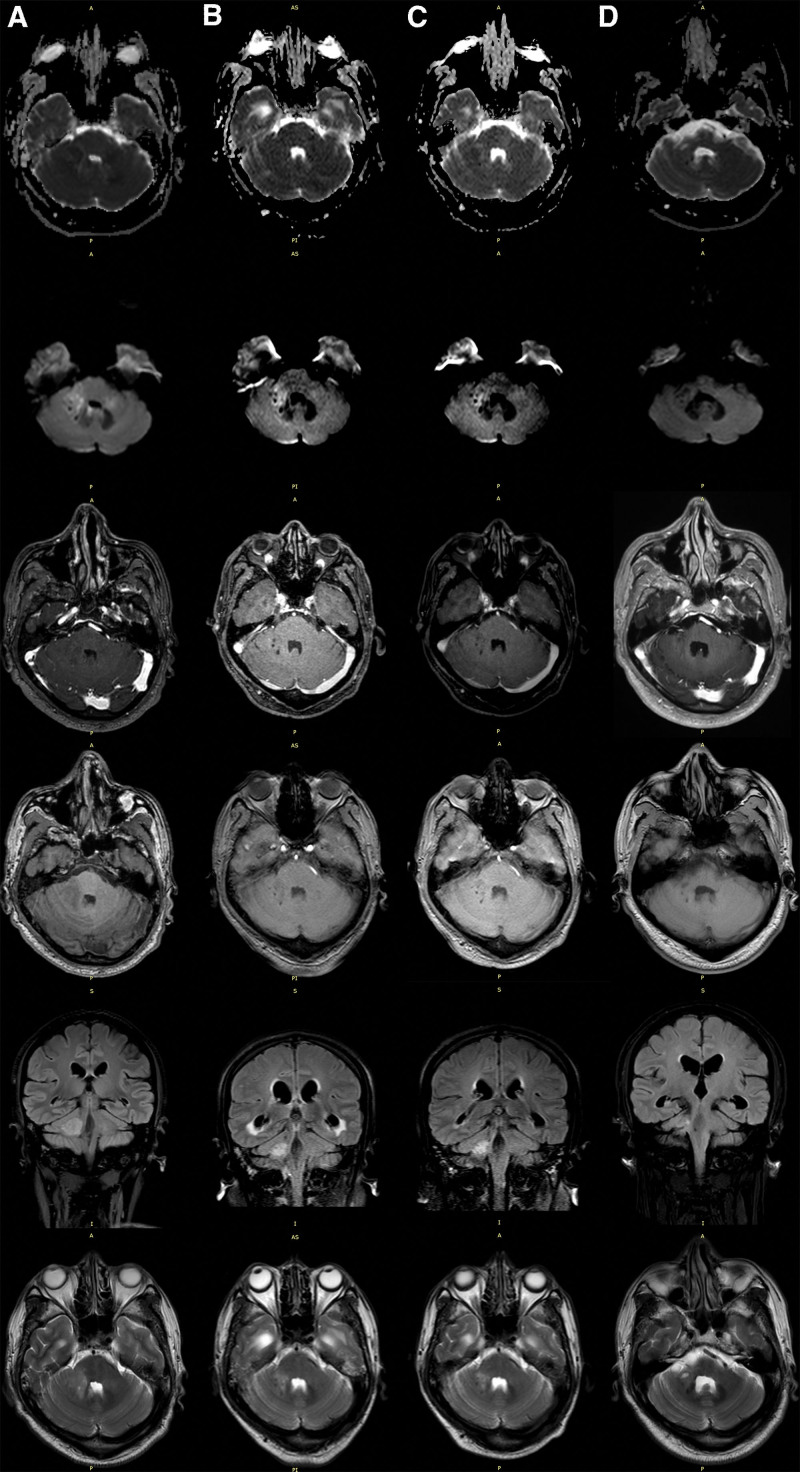
MRI of the head process. From top to bottom: DWI ADC, DWI b1000, T1W + C, T1W, T2 FLAIR, T2W. (A) After initial antibiotic treatment (September 14, 2022). The maximum length of the lesion on the right pons and brachium pontis is 2.9 cm, the maximum width of it is 2.5 cm, and the maximum circumference is 8.31 cm. The lesion signal: T1 slightly lower signal, with an average intensity of 222 (298); T2 high signal, with an average intensity of 463 (285); FLAIR high signal, with an average intensity of 711 (619); DWI slightly higher signal, with an average intensity of 352 (239); ADC slightly higher signal, with an average strength of 705 (654). The number of visible layers of the lesion is 4 (slice thickness = 5 mm). The lesion is not significantly enhanced but we can see local thickening of the left meninges with enhancement. (B) After 1 month Antibiotic Modification Treatment (October 11, 2022). The maximum length of the lesion is 2.5 cm, the maximum width of it is 2 cm, and the maximum circumference is 6.80 cm. The lesion signal: T1 slightly lower signal, with an average intensity of 168 (186); T2 high signal, with an average intensity of 628 (409); FLAIR slightly higher signal, with an average intensity of 374 (280); DWI slightly higher signal, with an average intensity of 351 (221); ADC high signal, with an average strength of 1116 (720). The number of visible layers of the lesion is 2 (slice thickness = 5 mm). The lesion is not significantly enhanced. The figure shows a significant widening of the supratentorial ventricle of the cerebellum. (C) On second admission (November 29, 2022). The maximum length of the lesion is 2 cm, the maximum width of it is 1.2 cm, and the maximum circumference is 4.94 cm. The lesion signal: T1 slightly lower signal, with an average intensity of 180 (199); T2 high signal, with an average intensity of 645 (471); FLAIR high signal, with an average intensity of 367 (263); DWI slightly higher signal, with an average intensity of 149 (138); ADC high signal, with an average strength of 807 (611). The number of visible layers of the lesion is 2 (slice thickness = 5 mm). The lesion is not significantly enhanced. The figure shows a significant widening of the supratentorial ventricle of the cerebellum. (D) On third admission (July 5, 2023). The maximum length of the lesion is 1.75 cm, the maximum width of it is 0.54 cm, and the maximum circumference is 1.91 cm. The lesion signal: T1 slightly lower signal, with an average intensity of 273 (356); T2 high signal, with an average intensity of 673 (359); FLAIR slightly higher signal, with an average intensity of 367 (284); DWI liquefaction necrosis low signal, with an average intensity of 57 (118), ADC high signal, with an average intensity of 1856 (906). The number of visible layers of the lesion is 1 (slice thickness = 5 mm). The lesion is not significantly enhanced. The figure shows a significant widening of the supratentorial ventricle of the cerebellum. Due to the lack of original MRI images in the external hospital 5 days before admission, we provide the description of the MRI result. The results indicate abnormal signals in the right pons and brachium pontis; the parentheses after the “average intensity” represent the average intensity of the surrounding tissue of the lesion, serving as a comparison.

**Figure 2.**
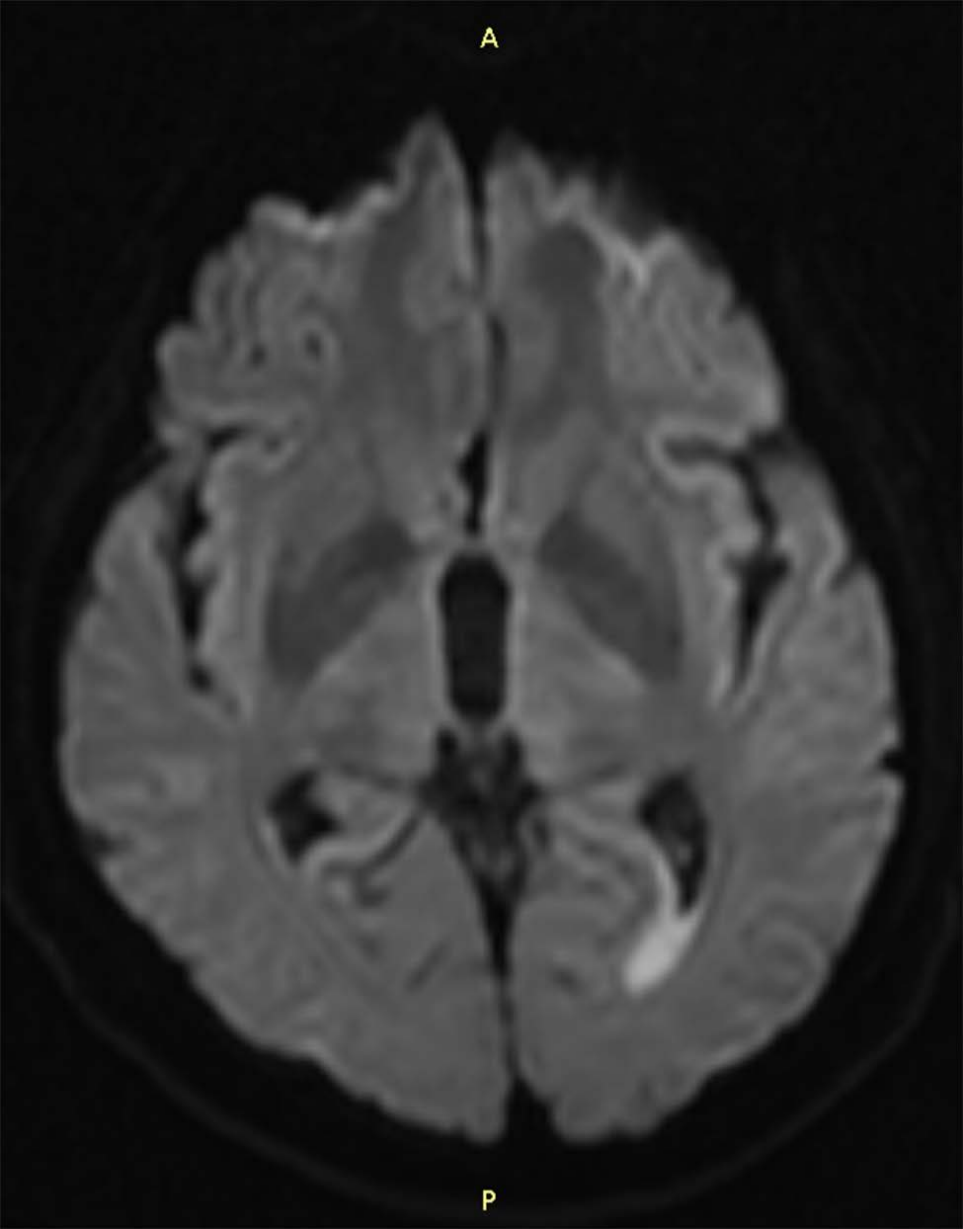
The lesion of the posterior horn of the newly developed lateral ventricle in DWI b1000 (September 14, 2022). A line segment is drawn from the farthest end of the posterior corner of the left lateral ventricle toward the farthest end of the lesion, with a length of 1.56 cm. The angle between the segment and the horizontal line is 60°. The maximum width of the lesion is 0.78 cm, and the maximum circumference is 7.98 cm. The signal of the lesion is high, with an average intensity of 319. The number of visible layers of the lesion is 2 (slice thickness = 5 mm).

**Figure 3.**
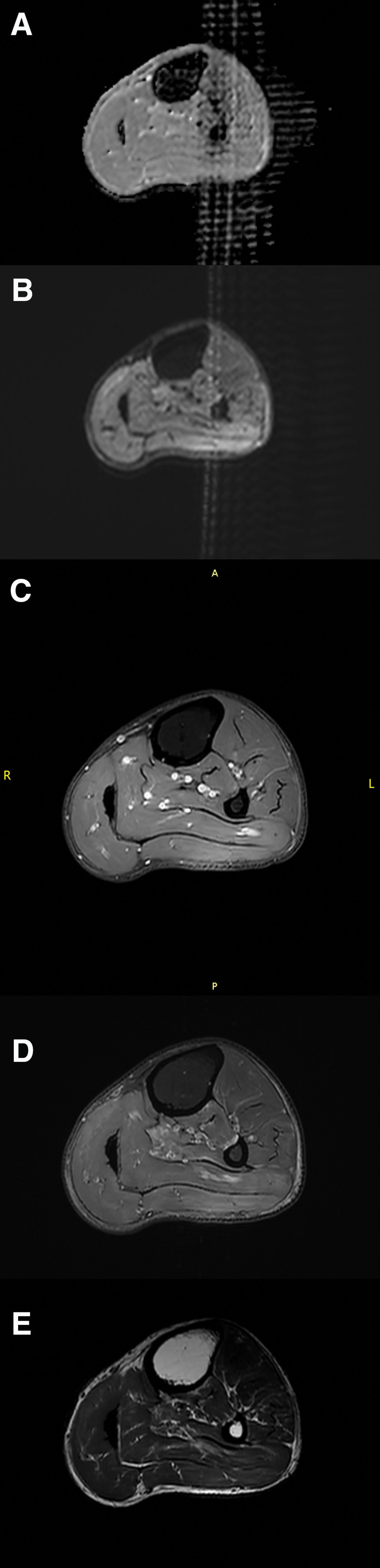
MRI of the muscle lesion (December 5, 2022). Left tibia and fibula and surrounding soft tissue MRI scan + enhancement: The surface of the left tibia and fibula is smooth, and there is no abnormal signal within the bone marrow cavity. There is no abnormal enhancement lesion. There is a high signal shadow in fat suppression (FS) of left tibiofibular posterior group muscle, especially the outer head of the gastrocnemius muscle. The diameter of the lesion is about 12.6 × 0.5 cm, and the internal vessels run naturally. (A) DWI TRA b600 L ADC. (B) DWI TRA b600 L NUC. (C) T1W FS TRA L + C. (D) T2W FS TRA L. (E) T2W TRA L. R = right, L = lift, A = anterior, P = posterior.

Half a year later, the reexamination showed only protein elevation in CSF and hydrocephalus in MRI. Laboratory examination can be found in Table 1, imaging examination can be found in Figures 1–3.

**Table 1:** Laboratory data.

Variable	5 days before current admission other hospital	On first admission this hospital	After initial antibiotic treatment	After 1 month antibiotic modification treatment	On second admission	On third admission	Reference range, this hospital^a^
Blood							
White-cell count (10^9/L)	19.52	7.1	6.7	3.3	4.7	5.7	3.5 to 9.5
Neutrophils count (10^9/L)	18.01	6.1	5.5	2.2	3.2	3.9	1.8 to 6.3
Differential count (%)							
Neutrophils	NA	86.6	81.5	67.7	67.4	69.1	40 to 75
Lymphocytes	NA	9.8	14.9	23.1	23.6	22.2	20 to 50
Monocytes	NA	3.6	2.8	5.9	6.4	6.7	3 to 10
Eosinophils	NA	0	0.8	2.5	2.3	1.6	0.4 to 8
Basophils	NA	0	0	0.8	0.3	0.4	0 to 1
Cerebrospinal fluid							
Pressure (mmH_2_O)	NA	60	60	125	70	85	80 to 180
Color	Colorless	Colorless	Yellow	Colorless	Colorless	Colorless	Colorless
Turbidity	NA	Relatively clear	Clear	Clear	Clear	Clear	Clear
Red-cell count (10^6/L)	0	0	0	0	0	0	<5
White-cell count (10^6/L)	210	1328	360	94	26	8	<8
Differential count (%)							
Neutrophils	NA	14.4	0.2	0	3.8	5	<6
Lymphocytes	NA	85.6	99.8	100	96.2	95	40 to 80
Protein (mg/L)	1371.5	4474.3	3285.1	1074.9	1379	1412.4	150 to 450
Immunoglobulin G (g/dl)	NA	425	264	73.7	99.5	115	4.8 to 58.6
Glucose (mol/L)	1.1	3.47	5.8	5.11	4.76	8.43	2.5 to 4.5
Chloride (mol/L)	NA	134.3	122.8	120.3	121.7	118.2	120 to 132

a Reference values are affected by many variables, including the patient population and the laboratory methods used. The reference ranges at the hospital in Drum Tower Hospital may therefore not be appropriate for all patients.

## 3. Discussion

We reported a rare case of severe LM meningoencephalitis complicated with muscle lesions, which may has some value for understanding *Listeria* infection.

In the early stage, the CSF showed a decrease in chloride and a moderate increase in cell count. Compared with other bacterial meningitis, the increase in cell count was not significant and could be misdiagnosed as tuberculous meningitis, cryptococcal meningitis, Lyme disease, and brucellosis. The common pathogenic identification features refer to Table 2 of Boulware et al.^[12]^ For the differential diagnosis of muscle lesions, it is necessary to distinguish them from traumatic, immunometabolic, tumor related and physiological myositis. First, the patient has not experienced any trauma, ruling out traumatic myositis. Second, the patient has no history of tumors, tumor markers are normal, and no tumors have been detected, thus ruling out tumor-associated myositis. Third, during the hospital stay, the patient did not engage in any intense physical activity, ruling out delayed onset muscle soreness and other physiological myositis. Fourth, autoimmune myositis often involves symmetrical proximal muscle, typically affecting the pelvic girdle and scapular girdle muscles, and is usually responsive to glucocorticoids. Our patient underwent glucocorticoids for a period of time, but there was no improvement in muscle symptoms or follow-up ultrasound. Therefore, the likelihood of autoimmune myositis is low.

**Table 2:** Typical features of common myositis.

Variable	Infectious (sepsis)	Traumatic	Tumor associated	Immunometabolic	Physiological
History of trauma	May have	Yes	No	No	No
Family history	No	No	May have	May have	No
History of tumor	No	No	Yes	May have	No
History of infection	Yes	No	No	No	No
Fever	May have	May have	May have	No	No
Age	No	No	Middle aged and elderly	No	Mainly young adults
Common sites	Lower leg muscles, followed by thighs	Four limbs	Pelvic girdle and scapular girdle muscles	Pelvic girdle and scapular girdle muscles	No
Muscle injury innervated by cranial nerves	May have	No	May have	May have	No
Symmetrical	Some	No	Yes	Some	Yes
Proximal/distal	Proximal or distal	Proximal or distal	Proximal	Proximal or distal	Proximal or distal
Muscle pain	Yes	Yes	Yes	Yes	Yes
Muscle atrophy	May have	May have	No	May have	No
Muscle weakness	May have	Yes	Yes	May have	May have
Skin damage	May have	Yes	May have	May have	No
Skin temperature	Sometimes increase	Increase or decrease	Basically unchanged	Basically unchanged	Basically unchanged
Sensation disorders	No	May have	May have	No	No
Damage to other systems	Yes	Yes	Yes	May have	No
Myoglobinuria	May have	Yes	May have	May have	May have
Decreased or absent tendon reflex	No	May have	Yes	May have	No
Creatine Kinase	Sometimes increase	Increase	Sometimes increase	Sometimes increase	Increase
Electrolyte disturbance	May have	Yes	May have	May have	No
Autoantibody test	No	No	Anti-3-hydroxy-3-methyl-CoA reductase protein (HMGCR) antibody, anti-NXP-2 antibody, anti-transcription mediator 1 (TIF-1) γ Antibody, etc	Antinuclear antibodies, myositis specific antibodies, anti-signal recognition particle (SRP) antibody, etc	No
Muscle MRI	Tissue edema, T2 weighted fat saturation (inhibition) sequence reveals enhanced signal throughout the leg muscles	The edge of the lesion has obvious edema, and there may be fibrosis and old hemorrhage	Muscle T2-weighted/fat-suppressed magnetic resonance imaging showed high intensity	Diffuse or patchy signal enhancement within muscle tissue, tissue edema or focal uneven muscle involvement	Can be negative, tissue edema, tear
Is cortisol effective	Mostly no	No	Yes	Yes	Yes, but it occasionally worsens

The patient in this case is more in line with the characteristics of infectious (sepsis) myositis.

The reason for the patient’s infection may be due to the patient’s accidental ingestion of spoiled or raw food in the refrigerator. Or it may be due to the patient’s previous digestive tract ulcer, promoting bacteria to enter the bloodstream. For patients suspected of LM infection, blood culture, NGS and CSF culture are crucial for diagnosis, and early use of sensitive antibiotics is key to improving prognosis and reducing mortality. However, it is worth noting that LM is naturally resistant to cephalosporins, and high-dose penicillin or ampicillin is recommended as a treatment for LM meningitis, with a replacement of Compound Sulfamethoxazole.^[13,14]^ The application of vancomycin and carbapenem in this disease has been controversial.^[15]^ And the course of antibiotic treatment is 2 to 4 weeks, and immunocompromised individuals can be appropriately extended to 6 weeks.^[16]^ Hormone therapy such as dexamethasone should be discontinue in bacterial meningitis caused by LM.^[17]^ However, we still use it intermittently, which may be one of the reasons for the long hospital stay of patients. Moreover, gastrointestinal bleeding occurred during the patient’s treatment period rather than during the onset of the disease. In addition, due to the genetic structural characteristics, the pathogenic mechanisms are mostly different. Therefore, identifying their unique complications or imaging features, or highly specific diagnostic markers, is helpful to early treatment.

LM can cause various complications in other systems,^[18]^ such as hepatic artery mycotic aneurysm,^[19]^ acute renal failure,^[20]^ pregnancy-related complications,^[21]^ etc. However, its concurrent muscle inflammatory lesions have never been reported. We speculate that LM may easily affect the muscle system, especially the flexor muscle groups, including tendons and muscles. Previously, no muscle lesions were found in LM infection, which may be masked by other symptoms, or it may have been treated as a comorbidity. We provide several possible explanations for why muscle lesions occurred in this case. First, bacteria entering the bloodstream can cause sepsis, and severe inflammatory factors can affect the muscles; Second, it produces autoantibodies that attack both LM antigen and muscle tissue; Third, it is estimated that muscle lesions are caused by the side effects and interactions of the drug, and the combination of moxifloxacin and dexamethasone increases the risk of tendinitis and tendon rupture.

The patient had low CSF pressure during several lumbar puncture procedures, which also caught our attention. Although the patient had hydrocephalus, we did not dehydrate the patient. This may also explain why the patient never experienced headache. In the early stage, it may be due to incomplete formation of meningeal adhesions and the fact that the lesion did not stimulate pain sensitive structures. While in the later stage, it may be related to a decrease in blood pressure after bleeding, as well as the use of dexamethasone in the course to alleviate adhesions.

The following suggestions for the comprehensive management of LM patients may be helpful. First, conduct multiple quantitative assessments of brain imaging and add muscle ultrasound screening. Second, use dehydration to reduce the incidence of refractory hydrocephalus in the later stage. Third, hormone use should be stopped in LM infection. Fourthly, in empirical treatments where culture and NGS results are not available, antibiotics should cover LM. If the following clinical manifestations and signs appear, LM meningoencephalitis should be highly suspected: onset in summer, history of unclean or refrigerated food consumption, or history of digestive tract lesions or surgery; acute onset, high fever with consciousness disturbance (drowsiness to light coma), positive meningeal irritation sign; white blood cell count (10–20) × 10^9, mainly neutrophils, with elevated or normal procalcitonin levels; clear or slightly cloudy appearance of CSF, white blood cell count (133–6666) × 10^6/L, CSF protein 0.6 to 7.6 [g/L], CSF/Serum glucose ratio (0.02–0.53), chloride levels normal or slightly reduced.^[22,23]^ For central nervous system infections in the elderly and immunocompromised population, the treatment effect for general bacterial meningitis is not satisfactory.

In conclusion, LM meningoencephalitis complicated with lower limb muscle lesions is clinically rare. This report focuses on relevant treatment plans, which provide certain value for the examination and comprehensive management of patients with LM infection in the future.

### 3.1. Patient perspective

The patient has never reported any symptoms of headache since the onset of the disease. As for the pain rating criteria of muscles, numerical rating scale (NRS) versions 0 to 10 were used.^[24,25]^ The patient rated himself as 3 points, indicating mild pain. The patient assessment of gastrointestinal disorders-symptom severity index (PAGI-SYM) scale was used to assess the symptoms of gastrointestinal tract.^[26]^ The patient complained of severe symptoms such as nausea and vomiting, with a score of 22 points at the onset. However, the score remained stable at 2 to 6 points after becoming conscious and experiencing relapse. This evaluation does not include situations such as gastrointestinal bleeding during the patient’s course of illness.

## Acknowledgments

We appreciate the Neurology Department of Nanjing Drum Tower Hospital for providing us with patient information. Multiple Disciplinary Team (MDT) was conducted in the comprehensive management, including Department of Neurology, Department of Neurosurgery, Department of Infectious diseases, Department of Gastroenterology, Department of Medical imaging, Department of Medical laboratory, Department of Orthopedics department. We also appreciate all participating consultation members for their contributions to the diagnosis and comprehensive treatment of the patient.

## Author contributions

**Formal analysis:** Xingbo Kou.

**Resources:** Xingbo Kou, Dinghao An.

**Writing – original draft:** Xingbo Kou, Dinghao An.

**Conceptualization:** Dinghao An.

**Data curation:** Dinghao An.

**Investigation:** Dinghao An.

**Validation:** Dinghao An.

**Writing – review & editing:** Dinghao An.

## Supplementary Material


