# Supplementary material for: A 48-year-old man with fever, nauseous, vomiting, and dizzy: A CARE case report
Source: Medicine (Baltimore). 2024 Aug 2;103(31):e39015. doi: 10.1097/MD.0000000000039015 (PMC11296406; doi:10.1097/MD.0000000000039015)
Supplement: Supplementary file 1 [file medi-103-e39015-s001.pdf]

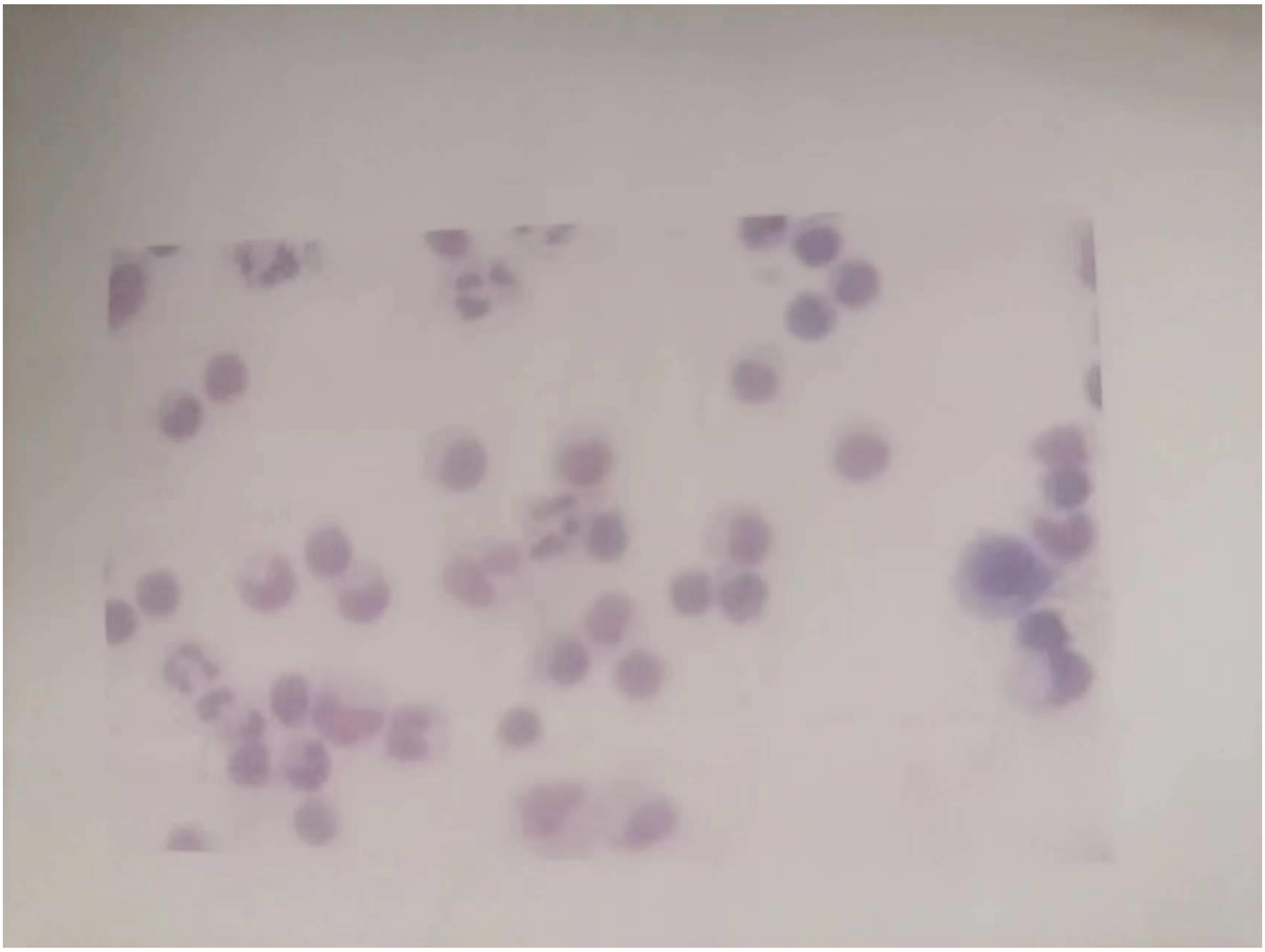

GensKey 金匙医学

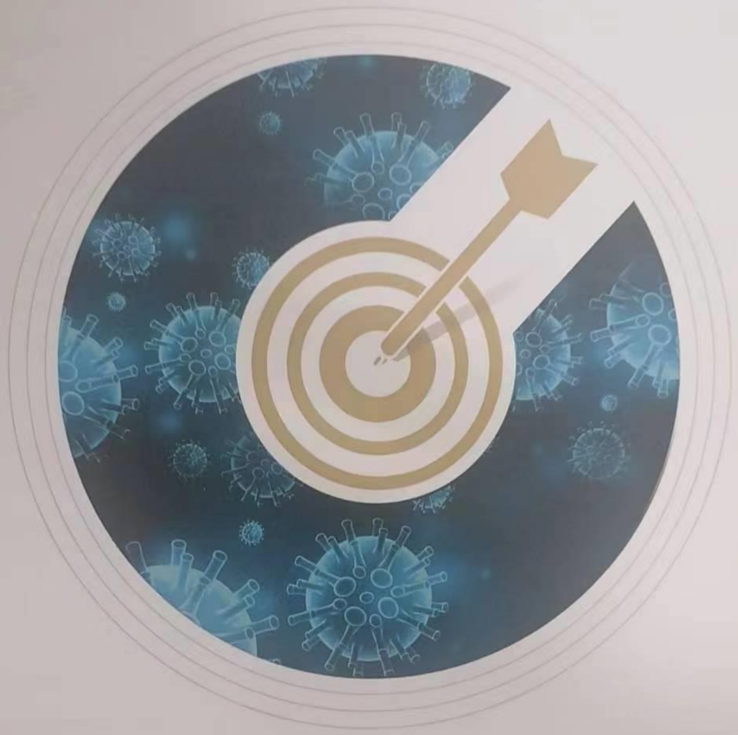

金识原® 感染病原体高通量测序

检测报告 TEST REPORT

患者姓名：王洪斌

报告编号：MBX123350

送检日期：2022-09-06

检测项目：金识原®病原微生物检测-DNA 检测

我们只服务于感染诊断事业

www.genskey.com

一、样品信息

受检者信息

**姓 名：	王洪斌	**性 别：	男	**年 龄：	48 岁
**电 话：	18251250521	住院号：	-	床 号：	-

样本信息

送检医院：	南京鼓楼医院	送检医师：	黄*医生	科室病区：	神经内科
采样日期：	2022-09-06	收样日期：	2022-09-06	报告日期：	2022-09-08
样本编号：	MBX123350	样本类型：	脑脊液		

临床信息

白细胞计数(WBC)： -	中性粒细胞比率： -
淋巴细胞比率： -	降钙素原（PCT）： -
C 反应蛋白 (CRP)： 30 mg/L	培养鉴定结果： -

主诉： 头晕伴恶心呕吐 20 天，发热 5 天

临床诊断： 中枢神经系统感染

临床高度关注病原类型： 支原体或衣原体,分支杆菌,寄生虫,真菌,病毒,细菌

抗感染用药及时长： 阿莫西林，克拉维酸钾 2 天

样本状态

颜色：无色　　性状：清晰透明无凝块

检测结果综述

细　菌： 单核细胞增生李斯特菌 2619 条，检出人体常见定植菌（具体见疑似微生态列表）

真　菌： 未检出疑似病原体，检出人体常见定植菌（具体见疑似微生态列表）

病　毒： 未检出疑似病原体

寄生虫： 未检出疑似病原体

样本编号：MBX123350 姓名：王洪[illegible]

二、DNA 检测结果

1. 疑似病原体检测结果

1.细菌列表

属				种	
类型	物种名	序列数	相对丰度(%)	物种名	序列数
G+	李斯特氏菌 *Listeria*	3412	95.63	单核细胞增生李斯特菌 *Listeria monocytogenes*	2619

2.真菌列表

未检出

3.病毒列表

未检出

4.寄生虫列表

未检出

5.结核分枝杆菌复合群列表

未检出

6.支/衣原体列表

未检出

病原解释说明及序列分布图*

1）**单核细胞增生李斯特菌** *(Listeria monocytogenes)*:

革兰阳性小杆菌，李斯特菌属。在自然界分布很广，可从环境如水、土壤、人和动物粪便、排水道和食品中分离出来，常伴随 EB 病毒引起传染性单核细胞增多症，也可引起脑膜炎、菌血症等。近年在发达国家常因污染奶制品而引起食物中度。

覆盖到基因组上的总长度为 131604 (bp),覆盖度为 4.4584% ,平均深度为 1.04 X。

样本编号：MBX123350　姓名：[redacted]

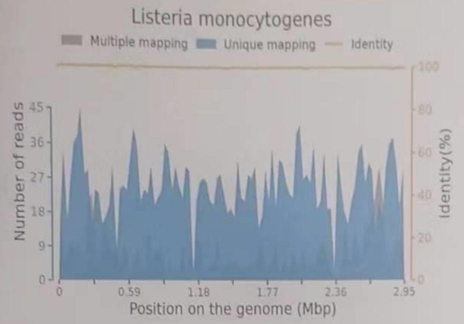

*序列分布图：该部分仅呈现检出非重复特异性序列≥3 条的物种序列分布图。

2. 细菌耐药基因检测结果

未检出

耐药基因检测结果说明：

1）可检测的耐药基因范围包括 mecA、VanA、VanB、ESBLs、AmpC、碳青霉烯酶基因等临床高度关注的耐药基因；
2）本检测通过将测序片段与已知耐药基因的序列进行比对，判断耐药基因是否存在，不适用于由于核苷酸突变引发的耐药；
3）抗生素的使用会促进耐药进程与变迁，同一患者不同时间采样耐药基因检测结果可能有差异；
4）耐药基因的基因型与实际耐药表型并不完全一致，所以报告检出的耐药基因仅供临床医生参考。

3. 细菌毒力基因检测结果

未检出

毒力基因检测结果说明：

1）毒力基因与疾病进程具有关联性，报告中检出的毒力基因仅供临床医生参考；
2）更多毒力基因信息可查看 http://www.mgc.ac.cn/VFs/.

4. 疑似微生态检测结果

疑似微生态列表	
属	种

样本编号：MBX123350 姓名：王洪斌

类型	物种名	序列数	相对丰度（%）	物种名	序列数
G+	丙酸杆菌属 *Cutibacterium*	20	0.56	痤疮丙酸杆菌 *Cutibacterium acnes*	15
G+	葡萄球菌属 *Staphylococcus*	19	0.53	沃氏葡萄球菌 *Staphylococcus warneri*	8
G+	葡萄球菌属 *Staphylococcus*	19	0.53	表皮葡萄球菌 *Staphylococcus epidermidis*	7
G+	棒杆菌属 *Corynebacterium*	13	0.36	拥挤棒状杆菌 *Corynebacterium accolens*	8
G+	微球菌属 *Micrococcus*	4	0.11	藤黄微球菌 *Micrococcus luteus*	4
fun	念珠菌属 *Candida*	2	2.02	近平滑念珠菌 *Candida parapsilosis*	2

★该病原在本次检测结果中相比其他微生态物种较突出，其属相对丰度占比 50%以上，单个菌种占属内一半以上，需重点关注。请结合临床症状考虑其致病可能。

疑似微生态物种解释说明

1）痤疮丙酸杆菌 *(Cutibacterium acnes)*:

革兰阳性杆菌，丙酸杆菌属。痤疮丙酸杆菌是皮肤上的优势菌群，栖居于毛囊、皮脂腺内，可从人的鼻腔、口腔、肠道和泌尿道中分离。此外，该菌是植入修复物或器械引起感染的主要病原菌，对原有心瓣膜损伤者可引起心内膜炎，是感染性心内膜炎的罕见病因。国外有报道该菌可引起中枢神经系统感染、眼部感染、口腔感染及呼吸系统感染。

2）沃氏葡萄球菌 *(Staphylococcus warneri)*:

革兰氏阳性的球形细菌，葡萄球菌属。主要存在于环境及人与动物的皮肤和粘膜上，有报道认为沃氏葡萄球菌与人类的动脉栓塞和牙周炎存在一定的关系。

3）表皮葡萄球菌 *(Staphylococcus epidermidis)*:

凝固酶阴性革兰阳性球菌，葡萄球菌属。表皮葡萄球菌是人体皮肤和黏膜上定居的正常菌群之一，为人类机会致病菌，通常情况下致病力很低。近几年来，随着留置静脉导管等侵袭性操作的增多，该菌可寄居在医疗设备上（如人工心脏瓣膜），引起术后感染及亚急性细菌性心内膜炎、腹膜炎、泌尿系感染、眼内炎、中耳炎及各类伤口感染等，已成为医院感染的重要致病菌。表皮葡萄球菌感染的诊断至今仍是一个难题，由于从实验的各个环节都有可能被污染，一般必须进行多次培养同时检出并结合临床才能作出诊断。

4）拥挤棒状杆菌 *(Corynebacterium accolens)*:

革兰阳性杆菌，棒状杆菌属。拥挤棒状杆菌分离自眼部、耳朵、鼻子和口咽等部位。由该菌引起的主动脉和二尖瓣心内膜炎已有详述。国外报道有中耳炎、结膜炎、脑膜炎、乳腺炎等，国内相关报道极少。

5）藤黄微球菌 *(Micrococcus luteus)*：

革兰阳性球菌，微球菌属。主要存在于泥土、水等外界环境以及人和动物的皮肤表面。一般不致病，在自然界和临床标本中较为常见，需要根据标本来源、菌落数量、菌落是否生长在接种线上等因素综合判断区分定植菌、污染菌还是感染菌。当人体免疫力低下时可引起各种机会感染，如菌血症、脑膜炎、心内膜炎等。

6）近平滑念珠菌 *(Candida parapsilosis)*：

念珠菌属，呈酵母样。近平滑念珠菌是一种常见的非白念珠菌，致病力较低，通常可以在皮肤、黏膜、外耳道等部位分离出，临床常难以区分侵袭性感染与无症状定植，患者临床表现可从局部粘膜病变（局部过度增殖和侵袭性感染）到播散性感染（血源性感染）。在自然界分布广泛，可存在于健康人的黏膜表面、皮肤以及指甲中。身体皮肤表面是最主要的分离部位，可引起皮肤浅表感染、中耳炎、鼻窦炎、眼和泌尿生殖道感染。从感染人群来看，近平滑念珠菌是低体重新生儿最常见的血流感染病原体。近平滑念珠菌、拟平滑念珠菌、似平滑念珠菌合称为近平滑念珠菌复合群。

名词解释：

- 序列数：匹配到该病原体的序列数目，其多少与标本中病原体本身载量负荷、核酸提取量、人源序列比例有关。序列数越高，表示标本中检测到该病原体的可信度越高。
- 相对丰度：该微生物在整个标本中检测到的相同类型微生物中所占的比重，丰度越高表示其在相同类型微生物中所占的比例越高。
- 覆盖度：表示检测到的该微生物核酸序列覆盖到该微生物整个基因序列的比值，覆盖度高表示该微生物全基因组测到的比率高。
- Multiple Mapping：多重比对，能够比对到 2 个及以上物种的 reads。
- Unique Mapping：唯一比对，唯一比对到某个物种的 reads。

4. 检测结果说明

本方法与其它检测方法一样，有自身的检测能力和检测范围，检测结果中未报告的微生物不代表样本中一定不存在，其原因包括但不限于：

1）样本中人源核酸比例过高、被检病原体在样本中的载量低于产品检测限；
2）针对新发病原或罕见病原，数据库可能未收录；
3）采样前使用抗感染药物，会导致微生物含量降低，可能会影响病原检出；
4）样本运输条件不合适，导致核酸降解、造成样本损耗等；
5）由 RNA 病毒引起的感染，DNA 流程无法有效检出，请关注 RNA 流程检测结果；

能引起临床发热的原因有很多种（参考下表），其中感染性发热占比约为 40%，非感染性发热临床占比约 60%，请临床医生结合其他临床诊断和症状做综合判断。

发热性质	病因	疾病
感染性发热(~40%)	各种病原体（细菌、病毒、支原体、衣原体、螺旋体、立克次体和寄生虫等）	急慢性全身或局灶感染
非感染性发热(~60%)	血液病	淋巴瘤、恶性组织细胞病、噬血细胞综合征、急性髓系白血病、多发性骨髓瘤等
	变态反应及结缔组织病	风湿热、药物热、系统性红斑狼疮、皮肌炎、白塞病、强直性脊柱炎、自身免疫性肝炎、反应性关节炎、成人 Still 病等
	实体肿瘤	肝和中枢神经系统转移瘤、肾细胞癌、肝癌、结肠癌、胰腺癌等

样本编号：MBX123350 姓名：王洪斌

三、测序质量

质控参数		样本数据
实验质控	内参	合格
	核酸提取浓度（ng/μL）	24.40
	文库浓度（ng/μL）	45.40
	阴控	合格
	是否去宿主	否
数据质控	总数据量	54593446
	非人源序列数	915374
	Q30（%）	92.14

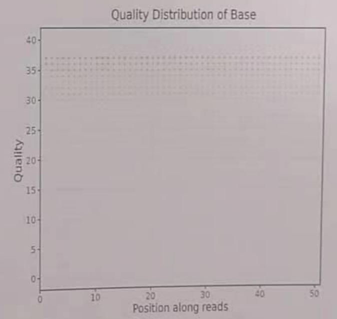

检测人：朱玉林　　审核人：陈莹　　报告日期：2022-09-08

天津金匙医学检验实验室 报告专用章

免责声明：
1）本报告结果只对本次送检样品负责。如有疑义，请在收到报告后的 7 个工作日内与我们联系；
2）以上检测结果仅供临床科研参考，不代表最终诊断意见；
3）因受检者知晓该结果可能带来的精神压力和心理负担，本检测机构不承担连带责任；

4）本检测对该结果保密并依法保护受检者隐私，但因受检者个人原因出现信息外泄，本检测机构不承担相应责任。

电子报告查询方式：

方式一：

微信扫码报告二维码，输入受检者姓名和预留手机号码，获取短信验证码后，即可查询；

方式二：

微信公众号搜索关注“金匙医学检验实验室”，点击“病原检测-宏基因组报告查询”，输入受检者姓名和预留手机号码，获取短信验证码后，即可查询。

四、方法学介绍

金识原®感染病原高通量检测产品基于样本中核酸进行检测，鉴定样本中存在的可疑致病微生物，可检测范围包括基因组序列已知的 12895 种细菌、11120 种病毒、1582 种真菌、312 种寄生虫、分枝杆菌复合群中的 177 种常见致病菌和 184 种支原体 / 衣原体。同时，检测鉴定样本中存在的疑似耐药基因，从而根据耐药基因预测样本中细菌的可能耐药性。本检测报告提供样本中可检出的所有具有有效数据的微生物，通过报告解读协助临床医师进行分析判断。适用于不明原因发热、疑难危重以及免疫缺陷等感染患者。

我们采用高通量测序技术，对样本中微生物核酸序列进行分析，通过与数据库中已有微生物的核酸序列进行比对，从而对微生物进行鉴定。以机器学习（machine learning）进行同步式错误探查（error modeling），背景清除（denoising）与精确序列推理（exact sequence inference），严格质控系统，自动消除假阳性结果。

技术流程

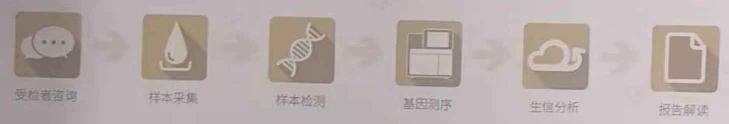

人体微生物分布

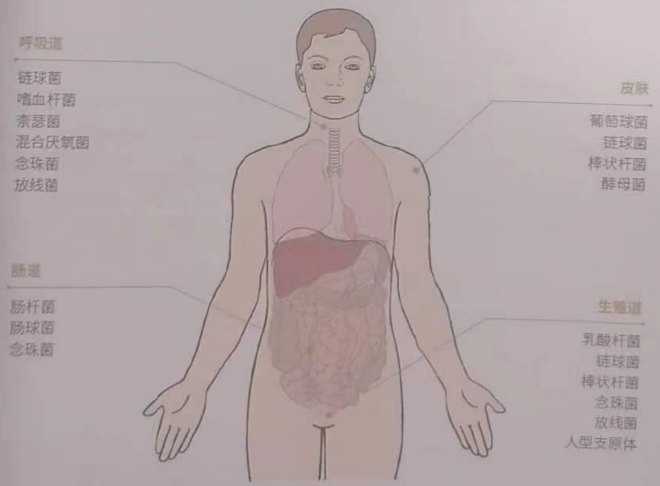

五、参考文献

1. 《临床微生物手册》：第 11 版 / (美) 詹姆斯 H. 约根森， (美) 迈克尔 A. 普法勒主编；王辉等译 .- 北京：中华医学电子音像出版社，2017.6
2. 《中华传染病杂志》编辑委员会 . 发热待查诊治专家共识 [J], 上海医学 ,2018.(41):385-400.
3. Blauwkamp TA, Thair S, Rosen MJ, Blair L, Lindner MS, Vilfan ID, et al. Analytical and clinical validation of a microbial cell-free DNA sequencing test for infectious disease. Nat Microbiol 2019;4(4):663-674.
4. Bouza E, Burillo A, Munoz P, Guinea J, Marin M, Rodriguez-Creixems M. Mixed bloodstream infections involving bacteria and Candida spp. J Antimicrob Chemother 2013;68(8):1881-8.
5. Claus RA, Otto GP, Deigner HP, Bauer M. Approaching clinical reality: markers for monitoring systemic inflammation and sepsis. Curr Mol Med 2010;10(2):227-35.
6. Decker SO, Sigl A, Grumaz C, Stevens P, Vainshtein Y, Zimmermann S, et al. Immune-Response Patterns and Next Generation Sequencing Diagnostics for the Detection of Mycoses in Patients with Septic Shock-Results of a Combined Clinical and Experimental Investigation. Int J Mol Sci 2017;18(8).
7. Grumaz S, Stevens P, Grumaz C, Decker SO, Weigand MA, Hofer S, et al. Next-generation sequencing diagnostics of bacteremia in septic patients. Genome Med 2016;8(1):73.
8. Guerrero-Latorre L, Romero B, Bonifaz E, Timoneda N, Rusinol M, Girones R, et al. Quito's virome: Metagenomic analysis of viral diversity in urban streams of Ecuador's capital city. Sci Total Environ 2018;645:1334-1343.
9. Manish Boolchandani, Alaric W. D' Souza & Gautam Dantas. Sequencing-based methods and resources to study antimicrobial resistance. Nature Reviews Genetics 2019; 20:356–370.
10. Jia et al. 2017. CARD 2017: expansion and model-centric curation of the Comprehensive Antibiotic Resistance Database. Nucleic Acids Research, 45, D566-573.
11. 宏基因组学测序技术在中重症感染中的临床应用共识专家组, 中国研究型医院学会脓毒症与休克专业委员会, 中国微生物学会微生物毒素专业委员会,等. 宏基因组学测序技术在中重症感染中的临床应用专家共识(第一版)[J]. 中华危重病急救医学, 2020, 32(05):531-536.
12. 《中华传染病杂志》编辑委员会. 中国宏基因组学第二代测序技术检测感染病原体的临床应用专家共识[J]. 中华传染病杂志, 2020, 38(11):681-689.

南 京 脑 科 医 院

南京医科大学附属脑科医院

病理报告单

送检医院：鼓楼医院

病理号：C20221966

姓名：王洪斌	性别：男	年龄：48岁	送检日期：2022-09-06
病区：	床号：	住院号：5161466	送检标本：脑脊液
临床诊断：中枢神经系统感染			染色方法：瑞氏+特染

外观：微黄

镜下：

白细胞：394 /ul

红细胞： /ul

分数：

中性粒细胞：	16	%
淋巴细胞：	72	%
单核细胞：	11	%
浆细胞：	1	%
吞噬细胞：		%
嗜酸性细胞：		%
嗜碱性细胞：		%
肥大细胞：		%

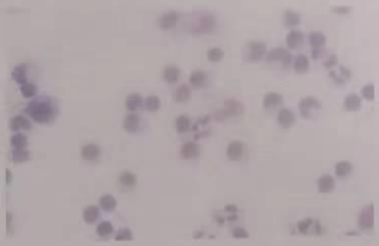

诊断意见：

重度中枢神经系统炎症，淋巴细胞为主性；未查见隐球菌及异型细胞。

报告仅供本院医师参考！仅针对本次检查！签名有效！

诊断医师：王娟 王娟

复诊医师：

报告日期：2022-09-06
